# PCSK9-antibodies fail to block PCSK9-induced inflammation in macrophages and cannot recapitulate protective effects of PCSK9-deficiency in experimental myocardial infarction

**DOI:** 10.3389/fcvm.2024.1463844

**Published:** 2025-01-21

**Authors:** Simon Rauterberg, Carmen Härdtner, Jennifer Hein, Paola Schrepf, Remi Peyronnet, Christoph Koentges, Tamara A. Vico, Carolin Ehlert, Bianca Dufner, Diana Lindner, Constantin von zur Mühlen, Dennis Wolf, Dirk Westermann, Ingo Hilgendorf, Alexander von Ehr

**Affiliations:** ^1^ Department of Cardiology and Angiology, Faculty of Medicine, University Heart Center Freiburg-Bad Krozingen, University of Freiburg, Freiburg, Germany; ^2^ Department of Congenital Heart Disease and Pediatric Cardiology, Faculty of Medicine, University Heart Centre Freiburg—Bad Krozingen, Medical Center—University of Freiburg, Freiburg, Germany; ^3^ Institute of Experimental Cardiovascular Medicine, Faculty of Medicine, University Heart Center Freiburg-Bad Krozingen, University of Freiburg, Freiburg, Germany; ^4^ Institute of Neuropathology, Medical Faculty, University of Freiburg, Freiburg, Germany

**Keywords:** PCSK9, PCSK9 inhibitors, alirocumab, PCSK9 deficiency, myocardial infarction, inflammation, macrophages

## Abstract

**Background and aims:**

Proprotein convertase subtilisin/kexin type 9 (PCSK9) plays a crucial role in cholesterol homeostasis by regulating low-density lipoprotein (LDL) receptor levels. Despite its known effects on cholesterol metabolism, the role of PCSK9 in cardiac function, especially post-myocardial infarction (MI), remains unclear. This study investigates the impact of PCSK9 on heart function post-MI and evaluates the effects of PCSK9 inhibition via Alirocumab.

**Methods:**

We used PCSK9 knockout (KO) mice and wildtype (WT) mice and *in vivo* treatment with Alirocumab to analyze cardiac function and survival post-MI induced by permanent ligation of the left anterior descending artery. PCSK9 and LDL receptor levels were measured using ELISA and qRT-PCR. Cardiac function was assessed via echocardiography and isolated working heart model experiments. Gene expression changes were evaluated using RNA sequencing, and inflammatory responses in bone marrow-derived macrophages (BMDMs) were analyzed *in vitro*.

**Results:**

PCSK9 was expressed in murine heart tissue at levels comparable to the liver, despite minimal heart RNA expression. PCSK9 KO mice had lower plasma cholesterol levels and showed reduced cardiac functions in the working heart model compared to WT mice. Post-MI, PCSK9 KO mice demonstrated significantly improved survival and reduced ventricular rupture compared to WT mice. Alirocumab treatment, while effective in lowering plasma cholesterol, did not replicate the survival benefits seen in PCSK9 KO mice and even worsened cardiac function post-MI. *In vitro*, PCSK9 induced significant inflammatory responses in macrophages, which were not mitigated by Alirocumab.

**Conclusion:**

PCSK9 accumulation in the heart post-MI contributes to adverse cardiac remodeling and inflammation. Genetic deletion of PCSK9 confers protection against post-infarct mortality, whereas pharmacological inhibition with Alirocumab fails to reproduce these benefits and may exacerbate cardiac dysfunction. These findings highlight the complex role of PCSK9 in cardiac pathology and caution against the assumption that PCSK9 inhibitors will necessarily yield cardiovascular benefits similar to genetic PCSK9 deficiency.

## Introduction

Proprotein convertase subtilisin/kexin type 9 (PCSK9) is a member of the proprotein convertase family and plays a pivotal role in cholesterol homeostasis (1, 2). PCSK9 regulates low-density lipoprotein (LDL) cholesterol levels by binding to hepatic LDL receptors (LDLR), promoting their lysosomal degradation, and thus reducing LDLR availability for clearing LDL cholesterol from the bloodstream (3, 4). Gain-of-function (GOF) mutations in the PCSK9 gene have been identified as a genetic cause of familial hypercholesterolemia, with numerous such mutations reported (5, 6), increasing the risk for atherosclerotic cardiovascular diseases such as coronary artery disease (CAD), cerebrovascular disease, and peripheral atherosclerosis.

The critical role of PCSK9 in cholesterol regulation has made it an attractive target for lipid-lowering therapies (7, 8). This has led to the development of PCSK9 inhibitors, including monoclonal antibodies (e.g., Alirocumab and Evolocumab) and small interfering RNAs (siRNAs) (e.g., Inclisiran), which block PCSK9 function or production. Clinical trials have demonstrated the efficacy of these PCSK9-targeting drugs in reducing serum LDL cholesterol levels significantly and preventing major adverse cardiovascular events.

Although the liver is the main source of circulating PCSK9, its expression has also been confirmed in other tissues, including the kidneys, brain, small intestine, colon and various cell types such as vascular smooth muscle cells (VSMCs), endothelial cells (ECs), and macrophages (2). However, the function of PCSK9 in the heart, especially in hearts suffering from ischemic injury, remains poorly understood. In previous studies, PCSK9 deficient mice showed a concentric left ventricular (LV) remodeling and a significant reduction of exercise tolerance without changes in systolic LV function (9). Da Dalt explained this HFpEF-like phenotype by potential cardiac lipotoxicity due to intracardial lipid accumulation in PCSK9 KO mice.

In patients with a history of heart failure and a recent acute coronary syndrome, treatment with the PCSK9-antibody Alirocumab did not lead to a reduction in cardiovascular events despite potent LDL cholesterol reduction. On the contrary, the rate of non-fatal myocardial infarctions was even higher in this subgroup of patients (10). This observation prompted our interest in investigating the role of PCSK9 in the injured heart and with PCSK9 antibody treatment in particular.

In this work, we profiled hearts of PCSK9-KO mice and tested their fate post myocardial infarction. We treated mice with the PCSK9-inhibitor Alirocumab, which increased hepatic LDLR expression and reduced cholesterol levels, and subjected them to experimental myocardial infarction, while antibody-bound PCSK9 accumulated in the heart. Mechanistically, PCSK9 induced, dose-dependent gene expression changes were explored in cardiomyocytes, fibroblasts and macrophages, identifying macrophages as key responder cells to PCSK9 surges. Notably, PCSK9 binding to antibodies did not block its pro-inflammatory effects in macrophages.

## Methods

### Cholesterol quantification

Total plasma cholesterol was quantified using a colorimetric assay (Diagnostic Systems, Holzheim, Germany).

### Western blot

A tissue sample (∼30 mg) was homogenized in RIPA lysis buffer (Santa Cruz Biotechnology), shock-frozen in liquid nitrogen and thawed on ice. After removal of cell debris by centrifugation total protein concentration was determined using a BCA Protein Assay (Thermo Fisher Scientific, Waltham, MA). 20–30 µg of Protein per sample were loaded for SDS-PAGE and blotted using the Trans-Blot Turbo Transfer System using nitrocellulose membranes (Bio-Rad Laboratories Ltd, Hercules, CA). Stain free blot acquisition was used for total protein normalization. Primary antibodies (Anti-mouse Cd36 1:1.000, Anti-mouse Gapdh 1:10.000, Anti-mouse Ldlr 1:1.000, Anti-mouse Lrp1 1:5.000, Anti-mouse Vldlr 1:1.000, all Abcam, Cambridge, UK) were incubated overnight at 4°C, secondary antibody (Anti-rabbit IgG HRP-linked, Cell signaling Technology, Denver, MA) for 1 h at room temperature. After multiple washing steps chemiluminescent substrate was added for 1 min. Proteins were quantified using ImageLab 3.1 (Bio-Rad Laboratories Ltd, Hercules, CA).

### RNA extraction from myocardial tissue

A tissue sample (∼30 mg) was excised with a scalpel and transferred to an ice-cold round-bottom FACS tube. One milliliter of Qiagen QIAzol Lysis Reagent was added, and the tissue was homogenized. The samples were incubated at room temperature, then centrifuged to pellet cell fragments. Chloroform was added to the supernatant. After phase separation by centrifugation, the upper phase was transferred to a new tube and mixed with an equal volume of 70% ethanol. RNA was isolated from the lysate using the Qiagen RNeasy Mini Kit following the manufacturer's protocol.

### RNA extraction from isolated cells

RLT Lysis Buffer from the Qiagen RNeasy Micro Kit was supplemented with 1% *β*-mercaptoethanol. The buffer was added to each well, the cells were detached using a cell scraper. The lysate was transferred to a reaction tube, mixed thoroughly, and shock-frozen in liquid nitrogen. After thawing on ice, an equal volume of 70% ethanol was added and Qiagen QIAshredder was used. RNA was isolated from the flow-through using the Qiagen RNeasy Micro Kit according to the manufacturer's protocol.

### qPCR

For reverse transcription of RNA, Thermo Fisher Scientific High-Capacity cDNA Reverse Transcription Kit was used according to the manufacturer's protocol. Quantitative real-time polymerase chain reaction (qRT-PCR) was employed for gene expression quantification using Thermo Fisher Scientific TaqMan Gene Expression Assays probes (CD36 Mm01135198_m1, IL-1β Mm00434228_m1, IL-6 Mm00446190_m1, LRP1 Mm00464608_m1, PCSK9 Mm01263610_m1) and the qPCRBIO Probe Mix Lo-Rox buffer system. *β*-Actin was utilized as an endogenous control for gene expression normalization. The reaction was carried out using the CFX96 Touch Real-Time PCR System. Data analysis was performed using CFX Manager 3.1 software. The ΔΔCt method was used to analyze the data, and genes with a Ct value greater than 40 were considered non-expressed.

### RNA sequencing

The isolated RNA of each sample was eluted with 14 μl of water, from which 4 μl of RNA were used for library preparation using the NEBNext Ultra II Directional RNA Library Prep Kit. The library was sequenced on a NextSeq instrument with 75 bp paired-end reads using NextSeq 500 High Output v2 kit (Illumina). At least 45 million reads were acquired from each bulk sample. Fastq files were transformed to gene counts using the Galaxy platform (https://usegalaxy.eu/). Adapters and end bases were trimmed with Cutadapt (Galaxy Version 1.16.5) with Phred score lower than 20. Trimmed reads with length shorter than 20 bp were discarded. The trimmed reads were aligned to the human genome (hg38) using RNA STAR (Galaxy Version 2.7.2b) with default settings. FeatureCounts (Galaxy Version 1.6.4 + galaxy1) was applied to count the features from the forward stranded bam files. The gene count files were downloaded and imported into R (Version 4.3.1) for downstream analyses. We utilized dplyr (Version 2.3.4) to compute the gene count files. Differentially expressed genes (DEGs) were called by DESeq2 (Version 1.40.2). Significantly regulated genes were defined as genes that increased or decreased significantly as opposed to the untreated control. Statistical significance of DEGs were defined as adjusted *p*-value < 0.05 post-Bonferroni correction. Pathway analyses were conducted using the online tool Enrichr. Further analyses were performed using the EnhancedVolcano package (Version 1.12.0) and the ComplexHeatmap package (Version 2.10.0).

### Isolated working heart model

The hearts of wildtype and PCSK9-KO mice were excised and immediately placed in ice-cold Krebs-Henseleit Buffer (KHB), consisting of (in mmol/L): 128 NaCl, 5 KCl, 1 KH2PO4, 1.3 MgSO4, 15 NaHCO3, 2.5 CaCl2, and 5 Glucose. Retrograde Langendorff perfusion was then conducted at 37°C with KHB at a perfusion pressure of 50 mmHg. After cannulation of the left atrium, the perfusion mode was switched to a working mode with a preload of 15 mmHg and afterload of 50 mmHg. Following an initial equilibration period, the hearts were perfused for 60 min with KHB supplemented with 0.4 mmol/L palmitate bound to 3% BSA. Aortic pressure changes were monitored using a Millar Micro-Tip pressure catheter (Millar Instruments, Houston, TX, USA) inserted into the aortic cannula. The aortic developed pressure was determined as the difference between systolic and systemic pressures. The rate-pressure product was calculated by multiplying the aortic developed pressure by the heart rate.

Aortic and coronary flows were measured by collecting flow from the afterload line and the effluent from the heart, respectively, with cardiac output defined as the sum of both flows. Cardiac work (ml*mmHg/min) was calculated as the product of cardiac output and aortic developed pressure per minute. Myocardial oxygen consumption (MVO2) was assessed by measuring the difference in oxygen concentration between pre- (arterial, aO2) and post-heart (venous, vO2) samples using a fiber-optic oxygen sensor (Ocean Optics, Orlando, FL, USA). Cardiac efficiency was then calculated as the ratio of hydraulic work to MVO2.

To measure palmitate oxidation within the same perfusion, the amount of ^3^H_2_O released from [9,10-^3^H] palmitate (specific activity, 500 GBq/mol) was determined. ^3^H_2_O was isolated from [9,10-^3^H] palmitate by mixing 500 µl of perfusate sample with 1.88 ml of chloroform/methanol (1:2 v/v) for a 15-min incubation, followed by adding 625 µl chloroform and another 15-min incubation. A 2 mol/L HCl/KCl solution was then introduced, mixed, and incubated for a minimum of 30 min to form polar and non-polar phases. An aliquot of 1.8 ml from the polar phase was transferred to another tube and mixed sequentially with 1 ml of chloroform, 1 ml of methanol, and 900 µl of HCl/KCl solution, with a 15-min incubation after each addition. After the final addition and an incubation period of at least 30 min, two 500 µl aliquots were collected from the upper layer for ^3^H counting. Palmitate oxidation rates were calculated from ^3^H_2_O production, with adjustments for dilution during the separation process. The isolated working heart model was performed as previously described (11).

### Echocardiography

The echocardiographic examination was conducted under light anesthesia with isoflurane (induction dose 3.5 vol.%, maintenance dose 2 vol.%). Images were acquired using the Vevo 3100 ultrasound system. For the parasternal long-axis view, the transducer and ultrasound table were adjusted to visualize the aortic valve, minimize foreshortening of the heart apex, and maximize the left ventricle's dimensions. The transducer was then rotated 90° for the parasternal short-axis view. The analysis was performed blinded using VevoLab 3.1.0 software. In the parasternal long-axis view, the contours of the left ventricle were traced during systole and diastole to determine the left ventricular areas. Assuming an ellipsoidal ventricle, the ejection fraction (EF) was calculated. The thickness of the left ventricular posterior wall (LVPW) was measured in the parasternal short-axis view at a height of 6 mm from the apex.

### Permanent ligation

Myocardial infarction was induced by permanent ligation of the left anterior descending artery (LAD). The experimental animals were anesthetized with an intraperitoneal injection of 100 mg/kg ketamine and 10 mg/kg xylazine. To compensate for perioperative blood and fluid loss, 10 ml/kg of isotonic 5% glucose solution was administered intraperitoneally. Following orotracheal intubation, pressure-controlled ventilation was performed. Anesthesia was maintained with 2 vol.% isoflurane. A left lateral thoracotomy was performed. The left anterior descending artery was identified under a microscope and ligated using a suture.

### Alirocumab treatment

Subcutaneous injections of 3 mg/kg body weight of Alirocumab or human IgG control antibody were administered, diluted in sterile water with a total volume of 100 μl per mouse. WT mice received a single or 4 weekly injections of 3 mg/kg Alirocumab or IgG1 control antibody.

### BMDM cultivation

Both murine femurs were extracted. The epiphyses were removed, and the bone marrow was flushed out with PBS. The cell suspension was filtered, and cell concentration was determined. The suspension was centrifuged and the pellet was resuspended in complete medium (RPMI 1640 with 10% FCS, 1% penicillin-streptomycin, 60 ng/ml M-CSF). Cells were plated at 1 million cells per well in 3 ml in a 12-well plate and incubated at 5% CO2 and 37°C. On day 3, the medium was replaced with fresh complete medium containing 30 ng/ml M-CSF. On day 5, the medium was replaced with 1 ml of starvation medium (RPMI 1640 without FCS), and stimulants were added.

## Results

### PCSK9 is expressed in the heart

**Figure 1:**
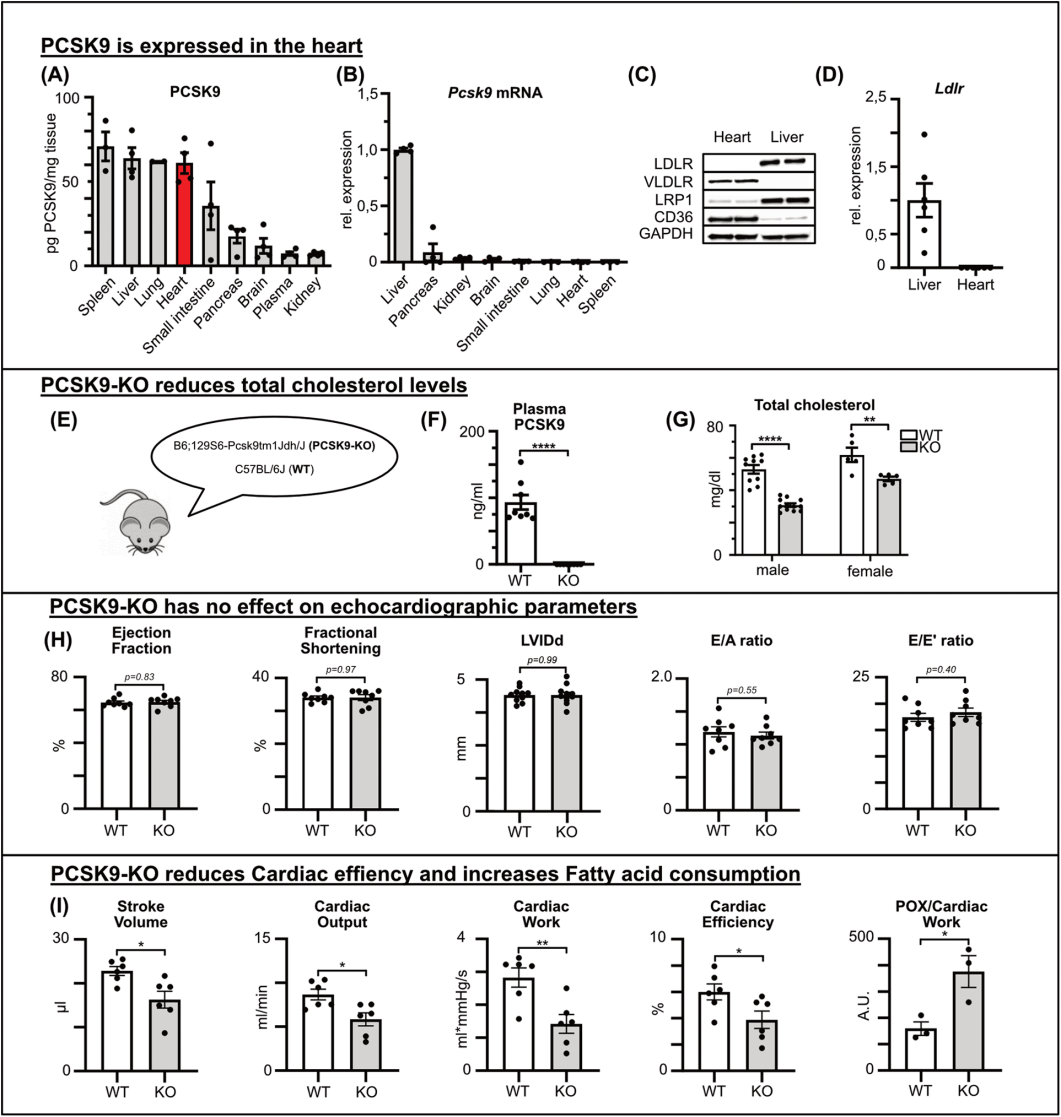
PCSK9 is expressed in the heart and influences cardiac functions PCSK9 protein concentration of different tissue and plasma lysates determined by ELISA normalized for total protein concentration determined by BCA assay **(A)** and *Pcsk9* RNA expression relative to hepatic expression **(B)** of C57BL/6 mice (*n* = 4). Using qRT-PCR, the expression of the LDL receptor **(D)** in the liver and heart was quantified (*n* = 6). **(C)** A representative Western blot illustrates potential PCSK9 targets in the heart and liver. PCSK9-ELISA **(F)** and cholesterol assay **(G)** of plasma samples from male and female C57Bl/6J wildtype (WT) and PCSK9-KO mice **(E)**, *n* = 8, **p < 0.01, ****p < 0.001 (*t*-test). No changes in echocardiographic parameters between WT and PCSK9-KO mice **(H)**. Results from Working heart model showing significant reduction in stroke volume, cardiac output, cardiac work, cardiac efficiency and fatty acid consumption/cardiac work. *n* = 8, *p < 0.05, **p < 0.01 (*t*-test) **(I)**.

To investigate the role of PCSK9 in the heart, the expression of PCSK9 and its binding partner LDLR were analyzed. PCSK9 expression in murine heart tissue was confirmed and quantified using ELISA, revealing comparable PCSK9 concentrations in heart and liver when normalized to total protein content (Figure 1A). However, at the RNA level, Pcsk9 expression was minimally detectable in the heart by qRT-PCR, indicating negligible on-site production (Figure 1B).

LDLR expression, a common binding partner of PCSK9, was detectable in the liver but not in the heart, as shown by qRT-PCR and Western blot analysis (Figures 1C,D). The expression levels of VLDLR, LRP1, and CD36, which have been proposed as alternative binding partners for PCSK9 (12–15), were explored by both Western Blot and qPCR of murine heart and liver tissue lysates. VLDLR and CD36 were predominantly expressed in the heart, while LRP1 was present in both organs (Figure 1C, Supplementary Figure A).

In summary, PCSK9 protein levels in the heart were relatively high despite negligible Pcsk9 RNA expression in the heart, suggesting that PCSK9 of plasmatic origin accumulated in the healthy heart even in the absence of the canonical binding partner LDLR.

### PCSK9 influences cardiac function

First, cardiac function was evaluated in healthy PCSK9 knockout (KO) mice compared with wildtype mice (Figure 1E). Genetic deletion was confirmed via ELISA, which showed undetectable plasma PCSK9 levels in KO mice, while wildtype mice exhibited baseline levels around 0.1 μg/ml (Figure 1F). In line with PCSK9 loss of function mutations in humans, PCSK9 KO mice featured significantly lower plasma cholesterol levels in both males and females compared to wildtype counterparts (Figure 1G).

Applying echocardiography under resting conditions, parameters such as ejection fraction, diastolic left ventricular inner diameter (LVIDd), and fractional shortening showed no differences between the genotypes. Likewise, diastolic function assessed by E/A and E/e‘ ratios was comparable between PCSK9 KO and WT mice (Figure 1H).

To investigate cardiac function in more detail, we used the working heart model *ex vivo* as previously described. In this model, PCSK9 KO mice demonstrated significant differences in key cardiac functional parameters, specifically decreased stroke volume, reduced cardiac efficiency and relatively increased fatty acid oxidation for comparable cardiac work as WT mice (Figure 1I).

**Figure 2:**
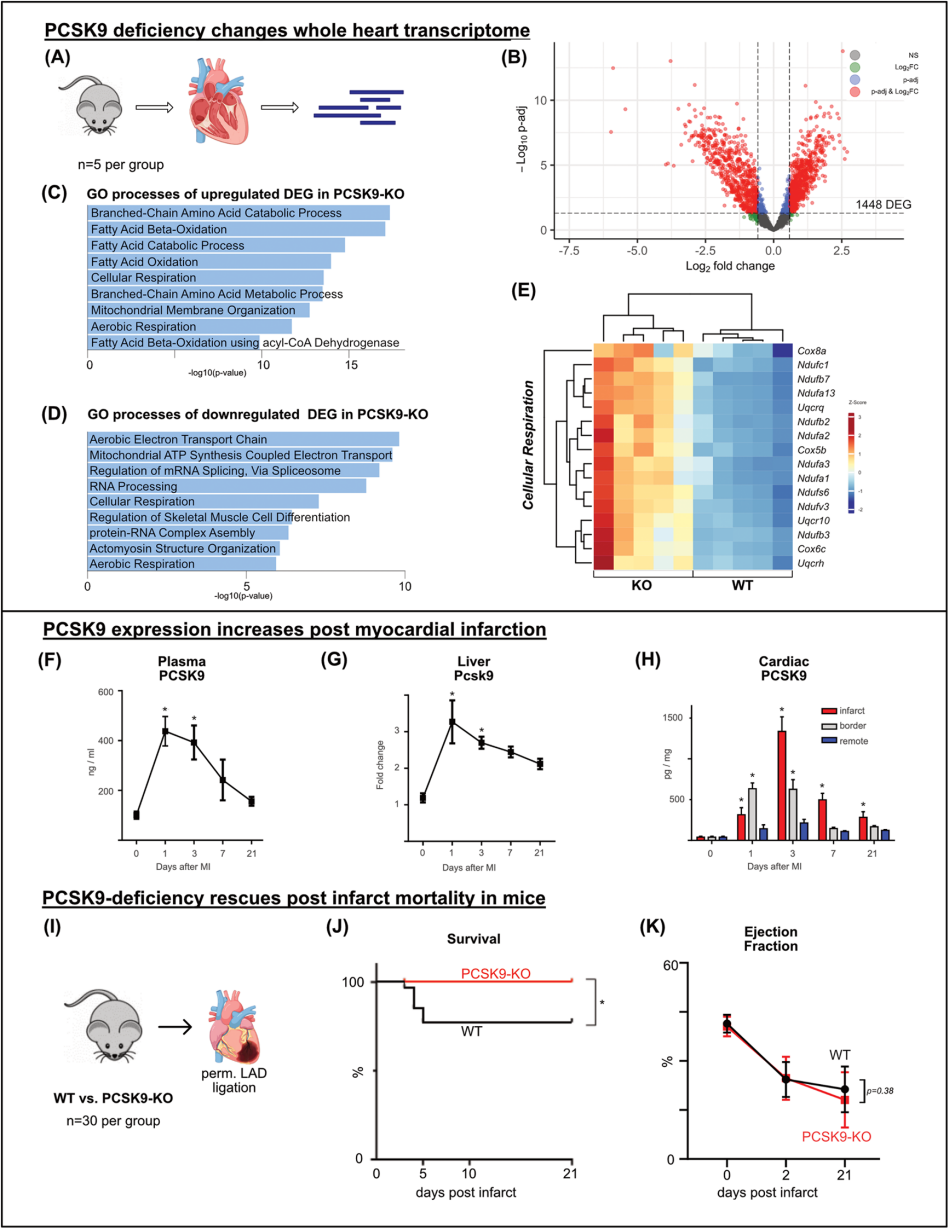
PCSK9 deficiency changes whole heart transcriptome and rescues post infarct mortality bulk-RNA sequencing of whole heart tissue was performed comparing the transcriptome of PCSK9-KO mice to WT, *n* = 5 per group **(A)** volcano plot of RNAseq results highlighting differentially expressed genes (DEG) (p-adj <0.05) in red and blue. A total of 1,448 genes were differentially expressed between the groups **(B)** Gene ontology analysis of up- **(C)** and downregulated **(D)** DEG separately using EnrichR webtool showing the top significantly altered gene ontology terms. Heatmap of DEG of the significantly enriched GO-term of cellular respiration **(E)**, *n* = 5 per group. Differential expression analyzed with DESeq2 tool. PCSK9-ELISA of plasma samples **(F)** and cardiac tissue lysates relative to total protein concentration determined by BCA **(H)** and qRT-PCR of liver samples relative to *β*-Actin expression **(G)** was performed at different timepoints following LAD ligation. Results are presented as mean ± SEM, *n* = 4−9 per timepoint, *p < 0.05 (One-way ANOVA, Dunnet post-test). Kaplan-Meier survival curve after permanent LAD ligation **(I)** showing significant survival benefit in PCSK9-KO mice **(J)**, *n* = 30 per group, *p < 0.05 (Log-rank test). No significant changes in ejection fraction at day 2 or 21 post MI **(K)** (Two-way ANOVA, Bonferroni's post-test).

To assess the underlying mechanisms of PCSK9's influence on cardiac function, we explored gene expression changes in the hearts of KO and WT mice. RNA sequencing revealed 1,448 differentially expressed genes (DEGs), with approximately half of the DEG being upregulated in KO hearts and the other half being downregulated (Figures 2A,B).

Gene ontology (GO) analysis using EnrichR was conducted separately for upregulated and downregulated DEGs. In line with the working heart model results, GO terms related to fatty acid consumption featured most prominently among the upregulated DEGs in KO hearts. Matching reduced cardiac work and efficiency in KO hearts, gene expression related to the aerobic electron transport chain and ATP synthesis was downregulated (Figures 2C–E).

PCSK9 appears to support homeostatic and metabolic cardiac function, but its loss can be compensated in the heart *in vivo* under resting conditions.

### PCSK9 levels rise post myocardial infarction

Permanent ligation of the left anterior descending artery (LAD) in mice was used as an experimental model for myocardial infarction. Animals were sacrificed before (day 0) and at 1, 3, 7, and 21 days post-surgery. PCSK9 concentration in plasma was determined by ELISA, showing a significant increase peaking at 0.4 μg/ml on day 1 post-infarction. Although the concentration gradually decreased thereafter, PCSK9 levels did not return to baseline for one week (Figure 2F).

In parallel, Pcsk9 gene expression in the liver, the main site of PCSK9 production, was analyzed using qRT-PCR. On day 1 post-infarction, liver Pcsk9 expression increased threefold from baseline, and then gradually decreased within one week mirroring the plasma PCSK9 levels (Figure 2G).

Myocardial tissue post-infarction was separated into three regions: the infarct area, a border zone region, and the non-infarcted remote area. These areas were lysed separately, and PCSK9 concentrations were measured using ELISA. At all post-infarction time points, a significantly increased PCSK9 concentration was detected in the infarct area, peaking on day 3. Elevated PCSK9 concentrations were also measured within the border zone during the first 3 days post infarction while the remote area showed only minor changed in PCSK9 accumulation (Figure 2H).

Taken together, myocardial infarction leads to a surge in PCSK9 concentrations in plasma, liver, and heart, suggesting a relevant role of PCSK9 in particular during the inflammatory phase of post MI cardiac remodeling.

### PCSK9-deficiency rescues post infarct mortality in mice

To evaluate the effect of PCSK9 on infarct healing, the permanent ligation model was applied to PCSK9-KO and WT mice. Between days 3 and 5, 20% of WT mice died, whereas all PCSK9-KO mice survived (Figures 2I,J). Autopsy revealed that ventricular rupture was the cause of death in all cases. Echocardiographic assessments were performed before, as well as 2 and 21 days after myocardial infarction. Among the surviving mice, no significant differences in cardiac function were observed between the genotypes as exemplified by drops in ejection fractions between days 2 and 21 post-infarction (Figure 2K). Our results indicate that the absence of PCSK9 has favorable effects on post-infarction survival and protection from ventricular rupture.

### PCSK9-inhibitor alirocumab fails to reproduce protective effects of PCSK9 deficiency in experimental myocardial infarction

**Figure 3:**
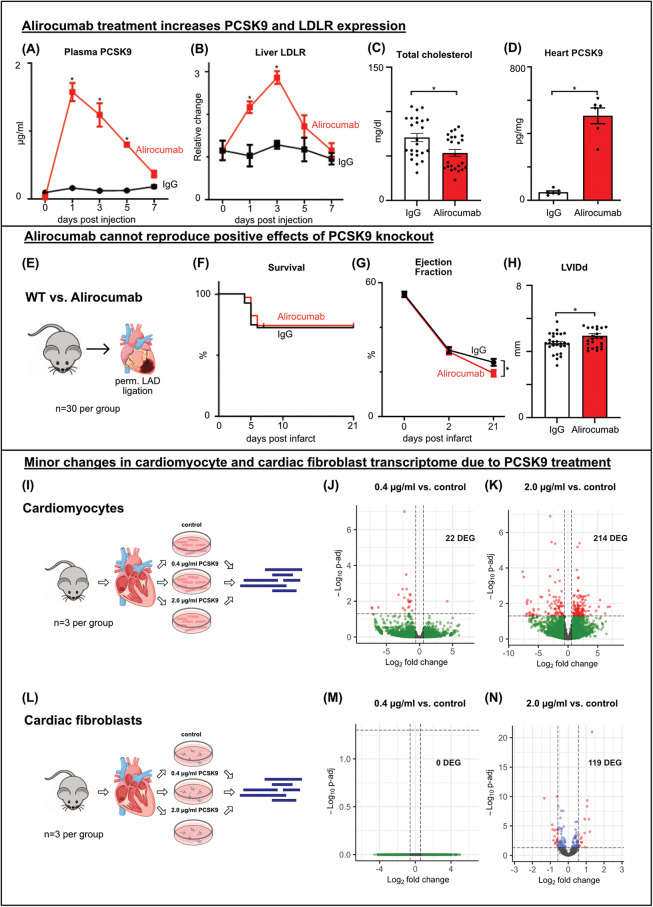
Alirocumab increases PCSK9 and LDLR expression and fails to reproduce protective effects of PCSK9 deficiency post MI WT mice received a single **(A, B)** or 4 weekly **(C, D)** injections of 3 mg/kg Alirocumab or IgG1 control antibody. Liver LDLR was analyzed using western blotting normalized for total lane protein **(B)**, plasmatic PCSK9 **(A)** and cardiac tissue lysate **(D)** PCSK9 normalized for total protein concentration using PCSK9-ELISA. Cholesterol assay of total cholesterol levels **(C)** *p < 0.05 Two-way ANOVA, Bonferroni post-test (A, B *n* = 3), *t*-test (C, D *n* = 24/6). WT mice received weekly subcutaneous injections of 3 mg/kg Alirocumab or IgG1 control antibody. LAD ligation **(E)** followed after 4 injections, echocardiography was performed before ligation (day 0) and 2 and 21 days after ligation **(G)**. Kaplan-Meier survival curves showing no differences between Alirocumab and IgG, *n* = 30 per group **(F)**. Left ventricular ejection fraction showing a significantly lower EF post MI in Alirocumab group, *p < 0.05, *t*-test (*n* = 25) **(G)**. LVIDd at day 21 post MI showing increased LV diameter after Alirocumab treatment **(H)**, *p < 0.05, *t*-test (*n* = 25). Results of Bulk RNA sequencing of cardiomyocytes **(I–K)** and cardiac fibroblasts **(L–N)** after *in vitro* stimulation with 0.4 µg/ml **(J, M)** or 2.0 µg/ml **(K, N)** PCSK9 compared to untreated controls (*n* = 3 per group). Volcano plots of DEG comparing PCSK9 treated cells to untreated controls. DEG are highlighted in red and blue (p-adj <0.05). Differential expression analyzed with DESeq2 tool.

To evaluate the translational implications of our findings in PCSK9 KO undergoing MI, we utilized the anti-PCSK9 antibody Alirocumab to inhibit PCSK9 *in vivo*. Wildtype mice received subcutaneous injections of 3 mg/kg Alirocumab or human IgG antibody as a control. PCSK9 targets the LDL receptor (LDLR) for degradation. Following a single Alirocumab injection, total murine PCSK9 levels surged in the plasma while being bound to the antibody (Figure 3A). As a result, hepatic LDLR concentrations peaked within three days, and returned to baseline around day 7 post injection (Figure 3B). These findings prompted us to use weekly Alirocumab injections in subsequent *in vivo* experiments. Four weekly injections of Alirocumab 3 mg/kg increased heart tissue concentrations by factor 10 compared to the IgG control group, while significantly decreasing plasma cholesterol levels (Figures 3C,D).

Next, Alirocumab or IgG pretreated mice underwent permanent LAD ligation to assess whether the survival benefit observed in PCSK9-KO mice can be recapitulated (Figure 3E). However, a comparable number of animals died post MI in Alirocumab and IgG treated mice. Echocardiographic evaluation even documented a more pronounced reduction in ventricular ejection fraction and increase in LVIDd in Alirocumab treated mice at day 21 post MI compared to controls (Figures 3 F–H).

In summary, therapy with the PCSK9 inhibitor Alirocumab fails to replicate the beneficial effects observed with PCSK9 knockout. Instead, following myocardial infarction in mice, treatment with Alirocumab even worsens cardiac systolic function.

### PCSK9 changes cardiomyocyte and cardiac fibroblast transcriptome only marginally

Given that outcomes post MI diverged between PCSK9-KO mice and Alirocumab treated mice, we hypothesized that the supernaturally increased total PCSK9 levels in the circulation and heart following anti-PCSK9 binding could have deleterious effects on cardiac cells. To test this hypothesis, we isolated and cultured primary cardiomyocytes and cardiac fibroblasts from healthy murine hearts. The cells were divided into three groups and treated with two different doses of PCSK9 *in vitro*. One group received 0.4 µg/ml PCSK9, representing the PCSK9 concentrations measured post-myocardial infarction (MI) in the plasma and in different areas of the heart, while another group received 2.0 µg/ml PCSK9, corresponding to the PCSK9 concentrations measured in plasma following weekly Alirocumab injections. The third group served as an untreated control. The cells were stimulated overnight and subsequently processed for RNA isolation and sequencing (Figures 3I,L).

Stimulation with 0.4 µg/ml PCSK9 had negligible effects on gene expression in cardiomyocytes and cardiac fibroblasts. Only 22 DEGs were observed in cardiomyocytes treated with 0.4 µg/ml PCSK9 (Figure 3J, Supplementary Figure C). Stimulation with high-dose PCSK9 (2.0 µg/ml) led to modest transcriptional changes (119 DEG in fibroblasts, 214 DEG in cardiomyocytes (Figure 3K, Supplementary Figures E,G). However, these DEG failed to enrich for distinct GO terms (Supplementary Figures D,F,H).

In summary, gene expression profiling suggests that cardiomyocytes and fibroblasts do not appear as primary target cells for MI-associated or antibody-mediated PCSK9 surges. Therefore, we redirected our attention to monocyte-derived macrophages which feature prominently in the infarct and border zone where PCSK9 accumulates.

### PCSK9 induces inflammation in BMDM

**Figure 4:**
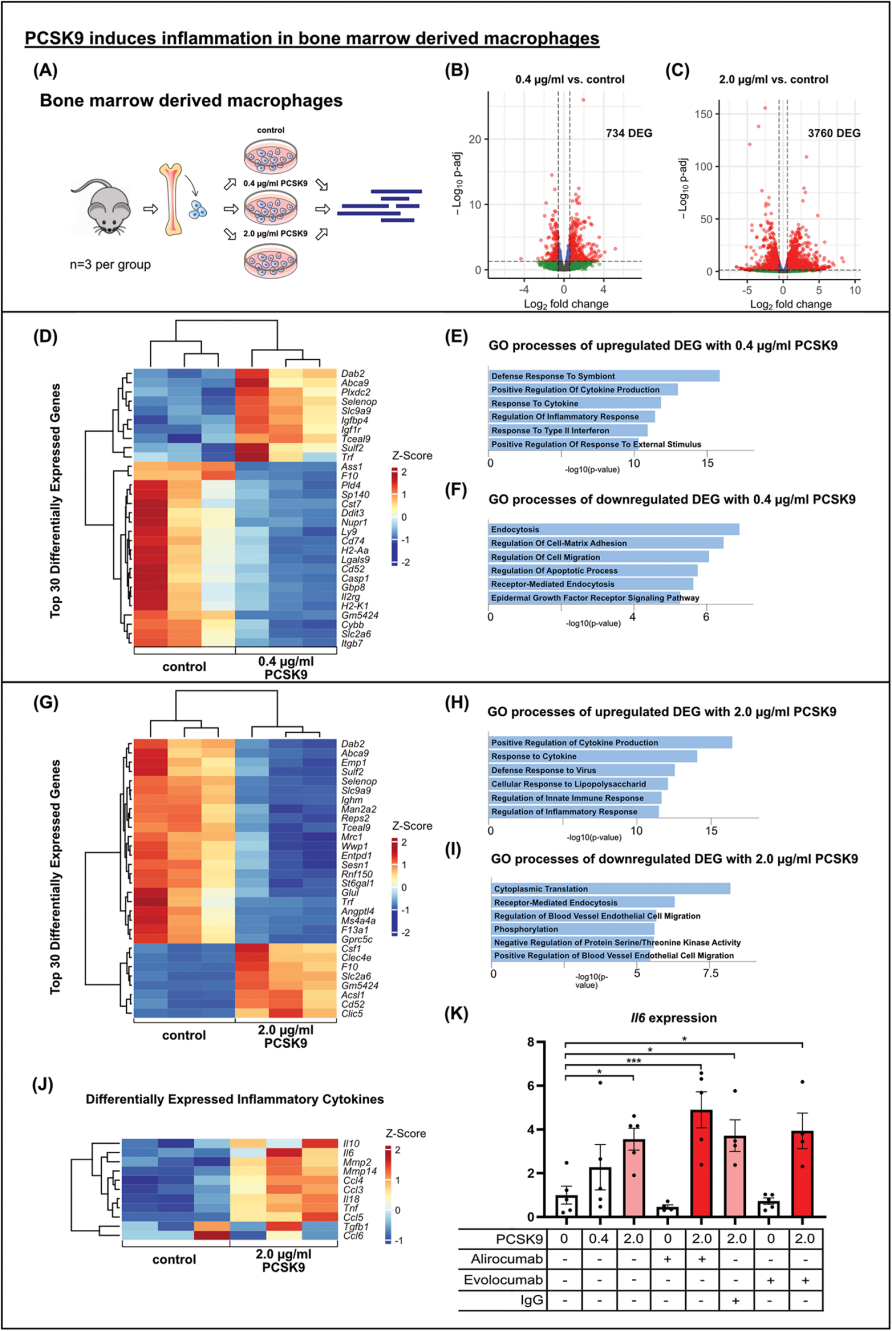
PCSK9 induces inflammation in bone marrow derived macrophages results of bulk RNA sequencing of bone marrow derived macrophages (BMDMs) after *in vitro* stimulation with 0.4 µg/ml **(B)** or 2.0 µg/ml **(A, C)** PCSK9 compared to untreated controls (*n* = 3 per group). Volcano plots of DEG comparing PCSK9 treated cells to untreated controls showing major changes in BMDM trancriptome due to PCSK9 stimulation. DEG are highlighted in red and blue (p-adj <0.05). Differential expression analyzed with DESeq2 tool. Heatmaps of the top 30 differentially expressed genes of each comparison (0.4 µg/ml PCSK9 vs. control **(D)** and 2.0 µg/ml PCSK9 vs. control **(G)** and DEG connected to inflammatory processes **(J)**. Gene ontology analysis of up- **(E, H)** and downregulated **(F, I)** DEG separately using EnrichR webtool showing significantly altered gene ontology terms. BMDMs were stimulated with different concentrations of recombinant mouse PCSK9, Alirocumab, Evolocumab and IgG control antibody overnight. IL-6 relative to b-Actin expression in macrophages (*n* = 5) using qRT-PCR **(K)**, *p < 0.05, ***p < 0.001 (One-way ANOVA, Dunnet post-test).

To this end, we generated bone marrow-derived macrophages (BMDMs) from WT mice and stimulated these cells *in vitro* with PCSK9 (Figure 4A). In contrast to our observations in cardiomyocytes and fibroblasts, stimulation of BMDMs with 0.4 µg/ml PCSK9 resulted in 734 DEGs, while the higher dose of 2.0 µg/ml PCSK9 yielded 3,760 DEGs (Figures 4B,C). The top 30 differentially regulated genes, ranked by adjusted *p*-value, are presented in heatmaps for the two doses (Figures 4D,G). In addition, GO terms enriched for up- or downregulated genes are presented separately for the two doses (Figures 4E,F,H,I). The 0.4 µg/ml dose induced genes associated with inflammation and cytokine production on the one hand, and suppressed genes related to endocytosis and migration on the other. The 2.0 µg/ml dose also induced many inflammatory genes including Interleukin 6 (IL-6) or tumor necrosis factor alpha (TNF) (Figure 4J). Quantitative RT-PCR analysis confirmed the dose-dependent induction of IL-6 gene expression in BMDMs. When Alirocumab was added to the BMDM culture together with PCSK9 at a dose sufficient to block PCSK9-mediated LDLR degradation in hepatocytes in culture (Supplementary Figure B), the IL-6 induction, however, was not ameliorated (Figure 4K). These results indicate that PCSK9 when bound to Alirocumab cannot facilitate LDLR degradation anymore but still induce inflammation in macrophages.

## Discussion

Our study provides significant insights into the role of PCSK9 and its inhibitors in the heart, particularly following myocardial infarction. Initially, we demonstrated the presence of PCSK9 in murine hearts. At the protein level, organ weight-normalized PCSK9 concentrations in the heart were similar to those in the liver, while *Pcsk9* RNA was hardly detectable in the heart, suggesting a dominant plasmatic origin of PCSK9 in the healthy heart. These findings are consistent with transcriptomic data from the human heart, which shows no expression of PCSK9 in various heart cells (16). Epicardial adipose tissue has been proposed as a potential source of PCSK9. In patients undergoing cardiac surgery, PCSK9 was detectable in epicardial adipose tissue at both RNA and protein levels, with its expression correlating positively with various inflammatory cytokines and the thickness of epicardial adipose tissue, but not with plasma PCSK9 concentrations, suggesting a local pro-inflammatory effect from the epicardial adipose tissue (17).The pathway through which PCSK9 exerts its effects in the heart remains unclear. Our study shows that the usual binding partner, LDLR, is not expressed in the heart. However, PCSK9 has been reported to interact with other receptors such as Low Density Lipoprotein Receptor-related Protein 1 (LRP1), Very-Low-Density-Lipoprotein Receptor (VLDLR), Apolipoprotein E Receptor 2 (ApoER2), and Cluster of Differentiation 36 (CD36) (12–15). VLDLR, LRP1, and CD36 were all detectable in murine hearts. These receptors are involved in cholesterol and fatty acid metabolism. For instance, PCSK9-deficiency in mice leads to CD36-dependent accumulation of fatty acids in hepatocytes, which can be cytotoxic (18).

Likewise, increased lipid accumulation was observed in hearts of PCSK9-KO mice (19). Da Dalt et al. described thickened ventricular walls and reduced exercise capacity with preserved left ventricular ejection fraction in 5-month old PCSK9-KO mice (9). In our study, we did not observe significant changes in echocardiographic parameters including diastolic function in PCSK9-KO mice at rest, whereby our mice were 2–3 month old at the time of examination. However, we did observe a reduction in stroke volume and cardiac work in the sensitive *ex vivo* working heart model, alongside inefficient myocardial fatty acid beta oxidation. In line with our observations in the working heart model and with the report by Da Dalt et al., we found genes controlling cellular respiration and mitochondrial ATP synthesis to be downregulated in PCSK9-KO hearts. These data confirm an effect of PCSK9 at physiologic levels on the healthy heart, which can be compensated functionally in young adult mice *in vivo*.

Myocardial infarction triggers a sterile inflammatory response locally and systemically, the balance of which is crucial for long-term heart function (20). Consistent with previous studies, we detected a significant and transient surge in PCSK9 concentrations in the plasma and infarct following permanent LAD ligation, accompanied by elevated expression in the liver (21, 22).

In our study, genetic deletion of PCSK9 in mice prevented ventricular rupture and death following experimental myocardial infarction. All PCSK9 knockout mice survived permanent LAD ligation, while 23% of wild-type animals succumbed between days 3 and 5 post MI. There were no differences in ejection fractions, a key parameter of cardiac function, between PCSK9-KO and WT mice at days 2 (before any WT mice died) and 21 post MI. Previous studies reported improved ejection fractions in PCSK9-KO mice following myocardial infarction but did not report any mortality (22, 23). The lack of EF differences on day 21 in our study likely reflects selection bias, as WT mice with larger infarcts and adverse remodeling were predisposed to ventricular rupture and thus excluded from late-stage analyses. Ventricular rupture is primarily driven by macrophage-mediated degradation of the interstitial network. Our studies indicate that PCSK9 exerts a strong pro-inflammatory effect in bone marrow-derived macrophages, including increased levels of IL-6, TNF, MMP2 and MMP14. In the absence of PCSK9 the inflammatory response post-MI may be reduced, resulting in lower risk of ventricular rupture and enhanced survival in the PCSK9-KO group. Reduced autophagy has also been proposed as a potential protective mechanism in PCSK9-KO mice post-MI (19, 22). In contrast, increased PCSK9 accumulation in the infarcted heart following PCSK9 antibody treatment will exert the opposite effect, given that PCSK9 remains biologically interactive with macrophages even when antibody-bound.

We observed minimal transcriptional changes in cardiomyocytes and cardiac fibroblasts cultured under normoxic conditions. In contrast, we and others detected an inflammatory response to PCSK9 in macrophages. Ricci et al. were the first to demonstrate that PCSK9 exerts a inflammation in macrophages *in vitro*. Recombinant human PCSK9 led to a dose-dependent increase in the expression of inflammatory cytokines IL-1β, IL-6, TNF-α, MCP-1, and CXCL2 in human macrophages derived from monocytes of healthy donors (24). Wang et al. reported that co-stimulation of RAW264.7 cells with LPS and recombinant murine PCSK9 resulted in higher IL-6 and iNOS expression compared to LPS stimulation alone (25).

In our study, we utilized macrophages derived from murine bone marrow cells (BMDM) as a model for recruited macrophages, stimulated with recombinant murine PCSK9 at different doses. Stimulation with 0.4 µg/ml PCSK9, representing peak plasma levels post MI, induced inflammatory pathways in BMDM, an effect further amplified with a stimulation of 2.0 µg/ml PCSK9. Prominent examples included the inflammatory cytokines IL-6, IL-18, and TNF.

Importantly, our study was the first to demonstrate that the pro-inflammatory effects of PCSK9 in macrophages were not inhibited by the blocking antibodies Alirocumab and Evolocumab. While Alirocumab was able to prevent the degradation of the LDL receptor in PCSK9-stimulated murine hepatocytes, it did not reduce the increase in IL-6 expression upon PCSK9 incubation. These results demonstrate that even antibody-bound PCSK9 can activate macrophages. Since we obtained similar results for both Alirocumab and Evolocumab, we do not attribute these effects to a specific therapeutic agent but rather view it as a class effect. This finding is significant because total PCSK9 levels in plasma, and specifically in the heart, driven by the surge of antibody-bound PSK9, multiply by a factor of 10 and more following repetitive Alirocumab injections in wild-type mice. Schroeder et al. reported similar results in mice expressing human PCSK9, attributing the accumulation of PCSK9 to inhibited degradation by Alirocumab (26). In patients, total plasma PCSK9 also accumulates above physiologic levels with Alirocumab treatment (27). Nakamura et al. administered Evolocumab or placebo to patients with acute myocardial infarction, finding that PCSK9 concentrations multiplied significantly after Evolocumab administration (28). In our study, we observed Pcsk9 accumulation in murine heart tissue following Alirocumab injections capable of exacerbating inflammation and deteriorating cardiac function post MI.

In light of these findings, it is noteworthy that therapeutic PCSK9 antibodies, unlike most cholesterol-lowering drugs, do not lead to a reduction in high-sensitivity C-reactive protein (hsCRP). A meta-analysis found that only the antibody LY3015014 resulted in a—albeit not significant—reduction in hsCRP levels (29). Unlike other antibodies, LY3015014 does not lead to an accumulation of PCSK9 in plasma (26). This finding might indicate a proinflammatory effects of accumulated PCSK9. These potential drug-specific effects are particularly relevant given alternative methods for inhibiting PCSK9. For instance, Inclisiran, which inhibits PCSK9 production via siRNA and reduces LDL cholesterol by 50%, does not lead to accumulation of PCSK9 and did reduce hsCRP inconsistently (30–32).

In conclusion, our study sheds light on a complex role of PCSK9 in the heart. At physiologic levels, mainly originating from the liver, PCSK9 appears to support homeostatic cardiac metabolism and function, although a loss of murine PCSK9 can be functionally compensated *in vivo*. In line, loss-of-function mutations in human PCSK9 were not found to be associated with an increased risk of heart failure in a large UK Biobank case-control study (33). Following myocardial infarction, however, PCSK9 levels exceed baseline levels by factor 4 to 10 in blood, the infarct and border zone, an effect further amplified by PCSK9-antibody mediated accumulation of PCSK9. Although effectively blocking PCSK9-mediated LDLR degradation in mice analogous to its effects in humans, the clinically approved PCSK9 inhibitor Alirocumab did not mitigate PCSK9-induced inflammation in macrophages. On the contrary, anti-PCSK9-mediated accumulation of PCSK9 aggravate adverse cardiac remodeling post MI in our study. This calls for a nuanced approach in clinical applications and a deeper understanding of PCSK9's multifaceted roles in cardiac physiology and pathology. As the landscape of PCSK9 inhibition continues to evolve, our study provides critical insights that could inform the development of more effective and safe therapeutic strategies for patients with cardiovascular disease.

## Data Availability

The original contributions presented in the study are publicly available. This data can be found here: https://www.ncbi.nlm.nih.gov/search/all/?term=PRJNA1209060.

## Supplementary material

Supplementary Figure S1 Relative expression of potential PCSK9 binding partners in heart and liver. mRNA levels were determined by qRT-PCR. Results are shown as mean ±SEM **(A)** Primary murine hepatocytes were isolated and stimulated overnight with varying concentrations of Pcsk9 and Alirocumab. The expression of the LDL receptor and *β*-actin as a control was analyzed via Western blot. Quantification of LDL receptor normalized to *β*-actin, (*n* = 3). Results are shown as mean ± SEM **(B)** Results of Bulk RNA sequencing of cardiomyocytes **(C-F)** and cardiac fibroblasts **(G, H)** after *in vitro* stimulation with 0.4 µg/ml **(C, D)** or 2.0 µg/ml **(E-H)** PCSK9 compared to untreated controls (*n* = 3 per group). Heatmaps of all **(C)** or the Top 30 DEG of each analysis **(E, G)**. DEG comparing PCSK9 treated cells to untreated controls. Differential expression analyzed with DESeq2 tool. Gene ontology analysis of DEG using EnrichR webtool showing the top significantly altered gene ontology terms **(D, F, H)**.
