# Supplementary figures and images for: PCSK9-antibodies fail to block PCSK9-induced inflammation in macrophages and cannot recapitulate protective effects of PCSK9-deficiency in experimental myocardial infarction
Source: Front Cardiovasc Med. 2025 Jan 21;11:1463844. doi: 10.3389/fcvm.2024.1463844 (PMC11790616; doi:10.3389/fcvm.2024.1463844)

Supplementary Figure 1

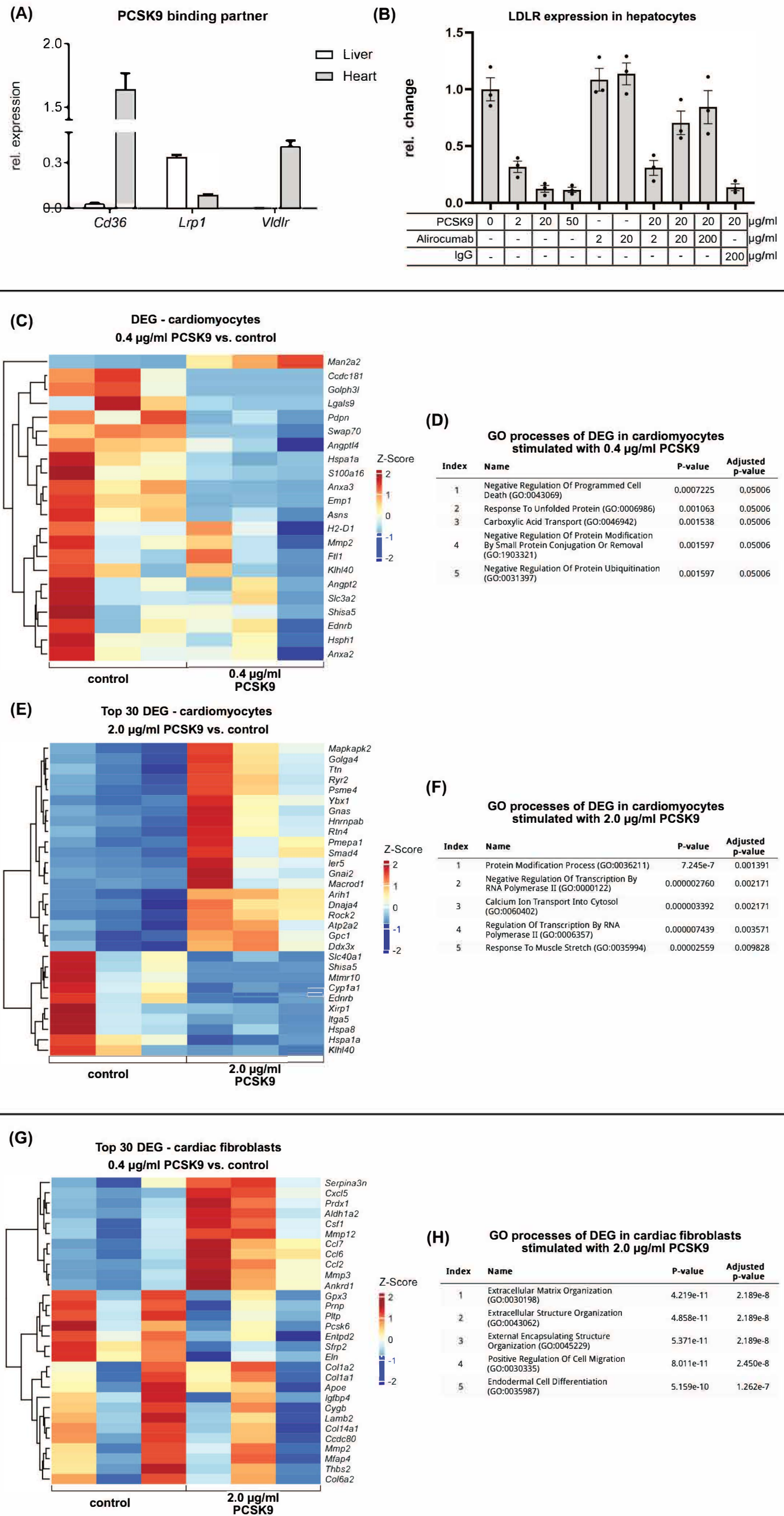

Supplement: Supplementary Figure S1 — Relative expression of potential PCSK9 binding partners in heart and liver. mRNA levels were determined by qRT-PCR. Results are shown as mean ±SEM (A) Primary murine hepatocytes were isolated and stimulated overnight with varying concentrations of Pcsk9 and Alirocumab. The expression of the LDL receptor and β-actin as a control was analyzed via Western blot. Quantification of LDL receptor normalized to β-actin, (n = 3). Results are shown as mean ± SEM (B) Results of Bulk RNA sequencing of cardiomyocytes (C-F) and cardiac fibroblasts (G, H) after in vitro stimulation with 0.4 µg/ml (C, D) or 2.0 µg/ml (E-H) PCSK9 compared to untreated controls (n = 3 per group). Heatmaps of all (C) or the Top 30 DEG of each analysis (E, G). DEG comparing PCSK9 treated cells to untreated controls. Differential expression analyzed with DESeq2 tool. Gene ontology analysis of DEG using EnrichR webtool showing the top significantly altered gene ontology terms (D, F, H). [file Image1.pdf]
